# Detecting cassava mosaic disease using a deep residual convolutional neural network with distinct block processing

**DOI:** 10.7717/peerj-cs.352

**Published:** 2021-03-02

**Authors:** David Opeoluwa Oyewola, Emmanuel Gbenga Dada, Sanjay Misra, Robertas Damaševičius

**Affiliations:** 1 Department of Mathematics and Computer Science, Federal University Kashere, Gombe, Nigeria; 2 Department of Mathematical Sciences, University of Maiduguri, Maiduguri, Nigeria; 3 Department of Electrical and Information Engineering, Covenant University, Ota, Nigeria; 4 Department of Computer Engineering, Atilim University, Ankara, Turkey; 5 Department of Applied Informatics, Vytautas Magnus University, Kaunas, Lithuania

**Keywords:** Cassava disease, Pattern recognition, Image processing, Deep learning, Convolutional neural networks, Distinct block processing, Data augmentation

## Abstract

For people in developing countries, cassava is a major source of calories and carbohydrates. However, Cassava Mosaic Disease (CMD) has become a major cause of concern among farmers in sub-Saharan Africa countries, which rely on cassava for both business and local consumption. The article proposes a novel deep residual convolution neural network (DRNN) for CMD detection in cassava leaf images. With the aid of distinct block processing, we can counterbalance the imbalanced image dataset of the cassava diseases and increase the number of images available for training and testing. Moreover, we adjust low contrast using Gamma correction and decorrelation stretching to enhance the color separation of an image with significant band-to-band correlation. Experimental results demonstrate that using a balanced dataset of images increases the accuracy of classification. The proposed DRNN model outperforms the plain convolutional neural network (PCNN) by a significant margin of 9.25% on the Cassava Disease Dataset from Kaggle.

## Introduction

Cassava or manioc is a starchy root vegetable or tuber. People in developing countries depend largely on it for consumption due to a large supply of carbohydrates. It grows in the world’s tropical regions because it can withstand harsh weather or unfavorable climatic conditions. The cassava roots are grown in many continents, such as Africa, Asia, and Latin America (Moses et al., 2008). In the tropical regions, more than five hundred million people, especially in Africa, rely on manioc as one of their main foods. Cassava production is mainly used for agriculture, as a feed for animals in both Asia and Latin America. In Africa, large quantities of manioc production are consumed as food by humans (Chikoti et al., 2019). The root is the most widely consumed component of cassava. It can be processed as bread, grated or grounded in meal, alcoholic beverage, whole, grated or grounded into flour or starch. In 2018, sub-Saharan cassava production is estimated to hit 161 million tons, or at a 2% higher than in 2017, according to the Food and Agriculture Organization (FAO, 2018). Cassava is susceptible to various diseases caused by viruses which include Cassava green mite (CGM), Cassava bacteria blight (CBB), Cassava brown streak disease (CBSD), Cassava mosaic disease (CMD), Cassava American latent disease (CALD), Cassava brown streak Uganda disease (CBSUD), Cassava Colombian symptomless disease (CCSD), Cassava frog skin-associated disease (CFSD), Cassava green mottle disease (CGMD), Cassava Ivorian bacilliform disease (CIBD), Cassava symptomless disease (CSD) and Cassava vein mosaic disease (CVMD) (Alabi, Kumar & Naidu, 2011). The most serious and common disease of cassava crop in Nigeria is CMD (Eni et al., 2018). The symptoms of CMD are varied including mottling, malformed and twisting of the leaf, and decrease in plant size. CMD prevents cassava plants from producing fruit, which results in financial loss and damage (Gautam & Kumar, 2020). It makes the plant produce little to no tubers at all depending on the extent of disease and plant age when they are infected (Maredza et al., 2016). To timely detect the occurrence of these diseases, Artificial Intelligence (AI) techniques are deployed in the context of smart farming (Farooq et al., 2019).

Recent studies have shown that deep learning-based approaches, which majorly use convolutional neural networks (CNN), were highly successful at image processing-related problems, based on its ability to extract efficient features from images (Polap & Wozniak, 2019; Urbonas et al., 2019; Capizzi et al., 2020; Khan et al., 2020), which excel the ability of classical algorithms such as Bag-of-Words (Gorecki et al., 2013; Gabryel & Damaševičius, 2017), dynamic time warping and Radon transform features (Santosh, Lamiroy & Wendling, 2011, 2013). Moreover, their result can be improved even more when coupled with heuristic optimization and evolutionary computing (Darwish, Hassanien & Das, 2020). Literature has shown that deep learning algorithms have gained wide acceptance among researchers and academicians for detecting different plant diseases (Mohanty, Hughes & Salathé, 2016; Sladojevic et al., 2016) such as banana (Amara, Bouaziz & Algergawy, 2017), tomato (Brahimi, Boukhalfa & Moussaoui, 2017), rice (Lu et al., 2017b), and citrus (Iqbal et al., 2018). This can be attributed to the efficacy of these algorithms in handling image segmentation and classification problems.

For example, Ramcharan et al. (2017) used cassava disease images to train a CNN to classify five diseases. The model accuracy of the best-trained model is 98% for brown leaf spot (BLS), 96% for red mite damage (RMD), 95% for green mite damage (GMD), 98% for CBSD, and 96% for CMD. A cumulative accuracy of 93% was reached by the best model. Their findings demonstrated that transfer learning techniques provide a quick, inexpensive, and accessible strategy for the detection of plant disease. Another smartphone-based CNN detection model (Ramcharan et al., 2019) achieved an accuracy of 94 ± 5.7% (mean ± s.d.), while Sangbamrung, Praneetpholkrang & Kanjanawattana (2020) achieved the accuracy and F-measure of 0.96 for the same dataset. Sambasivam & Opiyo (2020) used predictive machine learning models supported by image augmentation techniques to counter high-class imbalance and achieved an accuracy score of 93% in cassava disease detection. An image analysis technique for the detection of brown leaf spot caused in cassava was investigated by Abdullakasim et al. (2011). The authors used an artificial neural network (ANN) to classify between the healthy and infected plants. The algorithm correctly classified 79.23% of the disease leaves and 89.92% of healthy plants. Coulibaly et al. (2019) proposed a technique that applied transfer learning with feature selection to classify mildew disease in pearl millet. Deep learning was used by the authors to expedite a realistically quick and fascinating exploration of data in precision farming. The strength of their approach is that it has the potential to provide support to farmers for improved crop productivity. Ramcharan et al. (2017) applied deep CNN to detect some types of cassava diseases. The overall performance of their proposed method is good in terms of classification accuracy and confusion matrix. The drawback of their method is that the performance of the system was only evaluated against Support Vector Machine (SVM) and k-Nearest Neighbor (kNN). Moreover, only accuracy and confusion matrix were used as performance evaluation metrics in that work. These are not enough to truly validate the effectiveness and robustness of any classification technique. Lu et al. (2017a) proposed an active automated wheat disease recognition system based on supervised learning architecture. The performance of the proposed model is good in terms of accuracy and superior to that of traditional CNN models. The shortcomings of the proposed model are that a meager number of wheat disease images are contained in the database used for their experiments. Also, the only metric used to evaluate the model performance is accuracy which is not enough to validate the efficiency of the system. Ferentinos (2018) developed CNN models for the detection and diagnosis of plant disease. The dataset used contains about 87,848 images, having 25 distinct plants in a set of 58 separate groups of diseased and healthy plants. The advantages of the model include very high classification accuracy, and the ability to serve as a timely cautionary tool for farmers. The downside of the work is that other performance metrics were not used to validate the performance of the proposed model. Abayomi-Alli et al. (2020) used data augmentation techniques and generate synthetic images with modified color value distribution to expand the trainable image color space and to train the neural network to recognize important color-based features. Their approach is based on the convolution of the Chebyshev orthogonal functions with the probability distribution functions of image color histograms. Finally, the MobileNetV2 neural network is used for classification. Radial basis function neural network (RBPNN) for plant leaf disease identification and classification was proposed by Capizzi et al. (2016). Other classification methods such as Adaptive Artificial Neural Network (AANN) was introduced by Woźniak & Połap (2018). A comprehensive survey of image processing techniques used for leaf disease recognition was presented by Dhingra, Kumar & Joshi (2018). The summary of related work on plant leaf disease recognition is presented in Table 1.

**Table 1:** Summary of related work on plant leaf disease recognition.

Reference	Methods	Results
Abdullakasim et al. (2011)	Fully connected neural network (NN) with one hidden layer	79.23% of diseased leaves, 89.92% of healthy plants (accuracy)
Ramcharan et al. (2017)	Inception v3 convolutional neural network (CNN)	93% (accuracy)
Ferentinos (2018)	VGG CNN	99.53% (accuracy)
Ramcharan et al. (2019)	Single Shot Multibox (SSD) model with the MobileNet detector and classifier	94% ± 5.7% (accuracy)
Coulibaly et al. (2019)	VGG16 CNN	95.00% (accuracy), 90.50% (precision), 94.50% (recall), 91.75% (f1-score)
Sangbamrung, Praneetpholkrang & Kanjanawattana (2020)	Custom 15-layer CNN	0.96 (f-score)
Sambasivam & Opiyo (2020)	Contrast Limited Adaptive Histogram Equalization (CLAHE), Synthetic Minority Over-sampling (SMOTE), image flipping and custom 7-layer CNN	93% (accuracy)
Abayomi-Alli et al. (2021)	Color space augmentation and MobileNetV2 CNN	99.7% (accuracy)

We hereby propose a novel deep learning-based method that has the potential to overcome all the shortcomings notices in the techniques discussed above. Our novelty is the use of Deep Residual Convolutional Neural Networks (DRCNN) combined with distinct block processing for detection and classification of cassava mosaic disease.

The summary of contributions of this work are stated below:

- An overview of machine learning and deep learning algorithms that have been applied to cassava mosaic disease detection and classification is presented.
- A DRCNN model that surmounts the downsides associated with the existing methods that have been used for the detection, classification, and diagnosis of cassava mosaic disease is proposed.
- The proposed model is evaluated using different performance metrics and compared with plain convolutional neural network (PCNN) and other state-of-the-art algorithms.

The remaining parts of the paper are organized as follows. First, we describe the dataset used, the methods proposed for cassava leaves disease recognition, and the performance evaluation methods. Next, we present and discuss the results. Finally, we present conclusions.

## Materials and Methods

### Dataset

**Figure 1:**
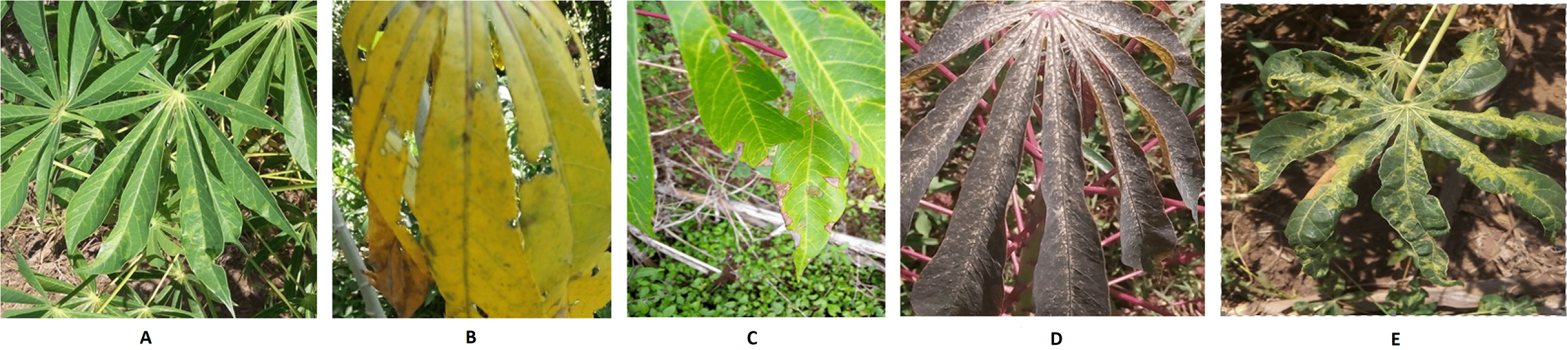
Images of healthy and unhealthy Cassava Mosaic Disease. (A) Healthy, (B) CBB, (C) CBSD, (D) CGM, (E) CMD Image credit: the Kaggle dataset at https://www.kaggle.com/c/cassava-disease.

**Figure 2:**
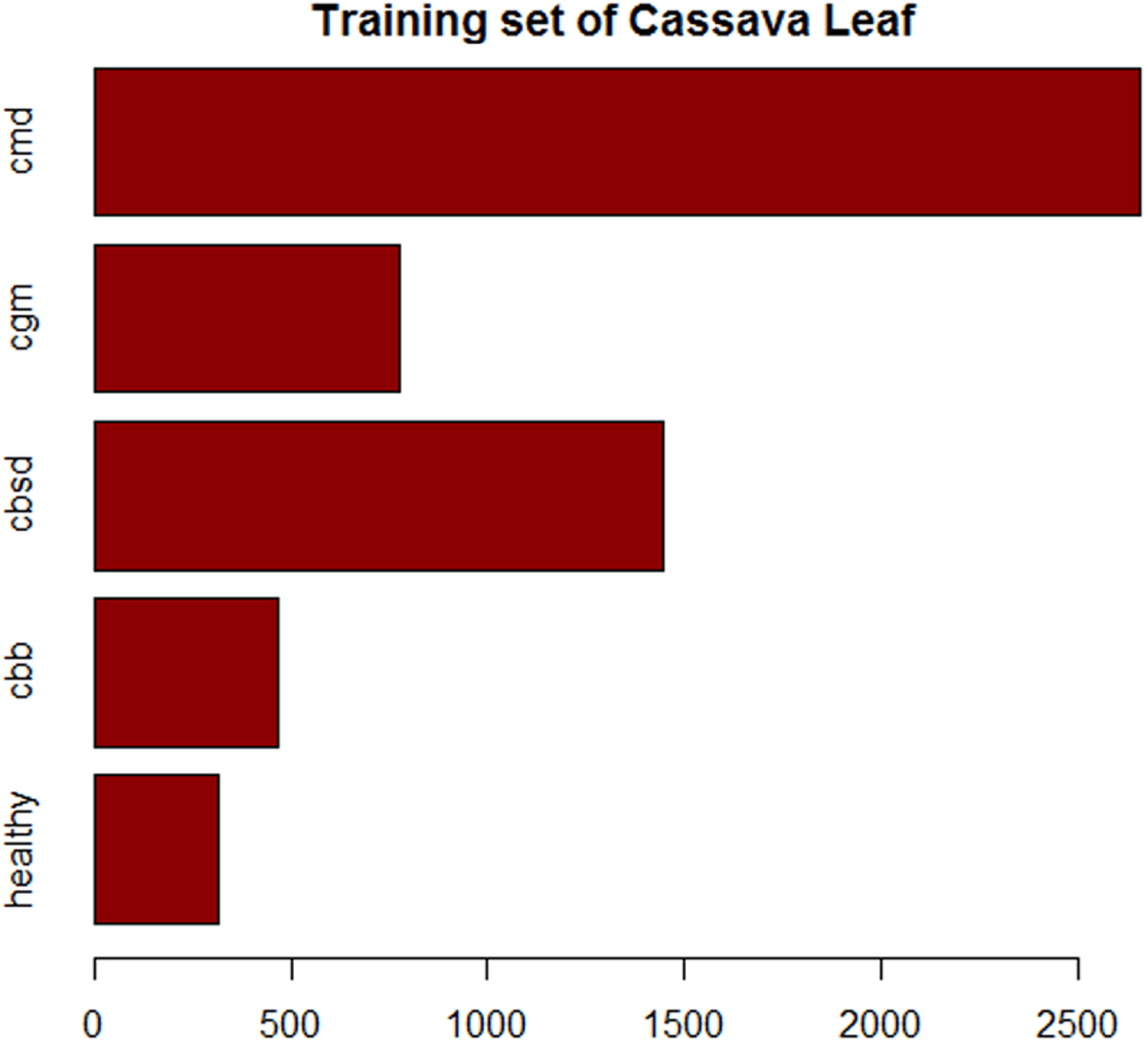
Composition of the training set of imbalanced cassava lead dataset.

**Figure 3:**
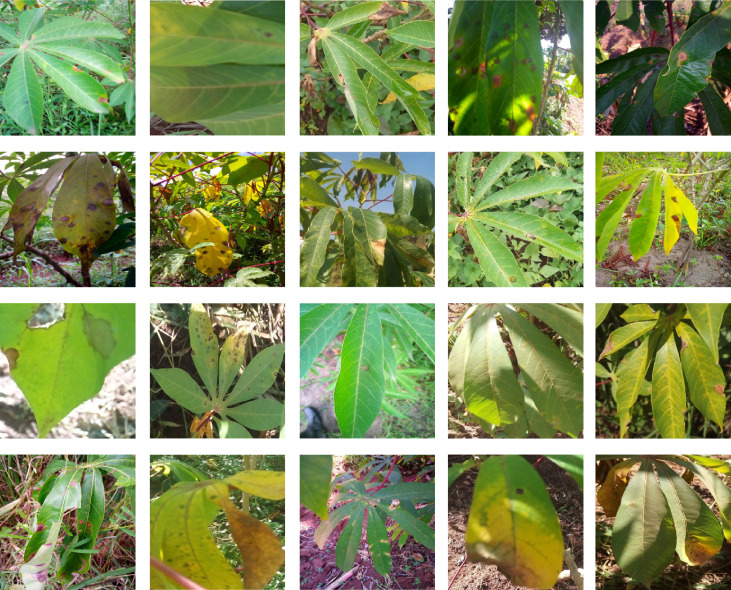
Examples of health and unhealthy cassava leaves. Image credit: the Kaggle dataset at https://www.kaggle.com/c/cassava-disease.

Images of cassava mosaic disease used in this research were obtained from the Kaggle database (Mwebaze et al., 2019). The dataset consists of 5,656 images with unequal instances of healthy cassava leaf (316) and four image sets of unhealthy cassava leaves: cassava bacteria blight (CBB) (466), cassava brown streak disease (CBSD) (1,443), cassava green mite (CGM) (773) and cassava mosaic disease (CMD) (2,658) obtained from farmers taking images of unhealthy cassava plants and annotated by experts as shown in Fig. 1. The number of cassava-healthy and unhealthy images in the dataset are shown in Fig. 2. Figure 3 is the random display of training images for both unhealthy and healthy cassava leaf disease. For our experiments, we use MATLAB ver 2019a. (Mathworks Inc., Natick, MA, USA).

### Proposed methods

Deep Convolutional Neural Networks (CNN) is used in this research to detect cassava diseases leaf. Instead of splitting the dataset into two classes: healthy and unhealthy classes. The dataset was split into five classes which are Healthy, CBB, CBSD, CGM, and CMD. The training dataset was very small in size and the classes were highly biased towards CMD, CBSD classes with low contrast and poor resolution. We improve on the low contrast using Gamma correction and decorrelation stretching to enhance the color separation of an image with significant band-to-band correlation. It also improves visual interpretation and makes feature discrimination easier.

The Gamma ${\rm \gamma}$ correction equation is given as:

(1) $${S_L} = \displaystyle{1 \over {\displaystyle{{\rm \gamma} \over {{B_p}^{\left( {\textstyle{1 \over {\rm \gamma}} - 1} \right)}}} - {\rm \gamma} {B_p} + {B_p}}}$$

(2) $${F_s} = \displaystyle{{{\rm \gamma} {S_L}} \over {{B_p}^{\left( {\textstyle{1 \over {\rm \gamma} } - 1} \right)}}}$$

(3) $${C_o} = {F_s}{B_p} - {S_L}{B_p}$$

(4) $$I = \left\{ {\matrix{ {{S_L}I,\; \; \; I \le {B_p}} \cr {{F_s}{I^{\rm \gamma}} - {C_o},\; I\;\gt\;{B_p}\; } \cr } } \right\}$$

where ${\rm \gamma}$ is gamma parameter, ${S_L}$ is the slope of the straight line segment, ${B_p}$ is the breakpoint of the straight line segment, ${F_s}$ is the slope matching factor, ${C_o}$ is the segment offset and $I$ is the input image.

### Distinct block processing

The imbalanced cassava mosaic disease dataset used in this paper is biased towards CMD and CBSD classes, and images have a different size. To address this issue, a distinct block processing technique was employed. Block processing is used when the resolution of input images is higher when the capacity of the neural network. Reducing the resolution would lead to information loss. Instead, block processing allows retaining information present in the images. Previously, it has been used successfully for various image classification tasks such as for segmentation of sono-mammogram images (Jothilakshmi et al., 2017) and image forgery detection (Al_Azrak et al., 2020).

In a distinct block operation, the input image is processed from block to block (Sharma et al., 2011). The images are split into rectangular blocks and operation is carried out individually on each block to determine the corresponding block image output and also specify the pixel values (Dubey & Jalal, 2014). Distinct blocks begin in the left upper corner, without overlapping the images.

Where the blocks do not fit the image, zero-padding was added to increase the number of images in less represented classes so that to have an equal number of images in all the five classes of cassava mosaic disease and resize all the images of the five classes of cassava mosaic diseases to the same size. Distinct block processing techniques were employed to increase each class to 2,700 images. The dataset increases to 13,500 images with equal instances of healthy cassava leaf (2,700) and four unhealthy cassava leaf images cassava bacteria blight (CBB) (2,700), cassava brown streak disease (CBSD) (2,700), cassava green mite (CGM) (2,700) and cassava mosaic disease (CMD) (2,700) classes.

### Model architecture

Convolutional neural networks are bio-inspired networks used to classify images and to detect objects (Rawat & Wang, 2017; LeCun, Bengio & Hinton, 2015). Every layer in the CNN is a 3D grid structure, with a height, width, and depth. The word “depth” refers to the number of channels in each layer, such as primary color channels, for example, blue, green, and red, in the input image or the number of hidden layers of the feature maps. The network works similar to feed-forward neural networks, except that convolutional layers are spatially structured (He et al., 2015; Mohanty, Hughes & Salathé, 2016). The three forms of layers mainly found in CNNs are convolution, pooling, and rectified linear units. The parameters are grouped into three-dimension structural components, identified as filters or kernels. The filters are normally spatially square. The dimensions on which the filter normally applies are far smaller than those of the filters. In formulating the model used in this research, the objective, methodology, and model architecture were taken into consideration. We considered two neural network models in this paper. The models are Plain Convolution Neural Networks (PCNN) and Deep Residual Neural Networks (DRNN).

### Plain convolution neural network

**Figure 4:**
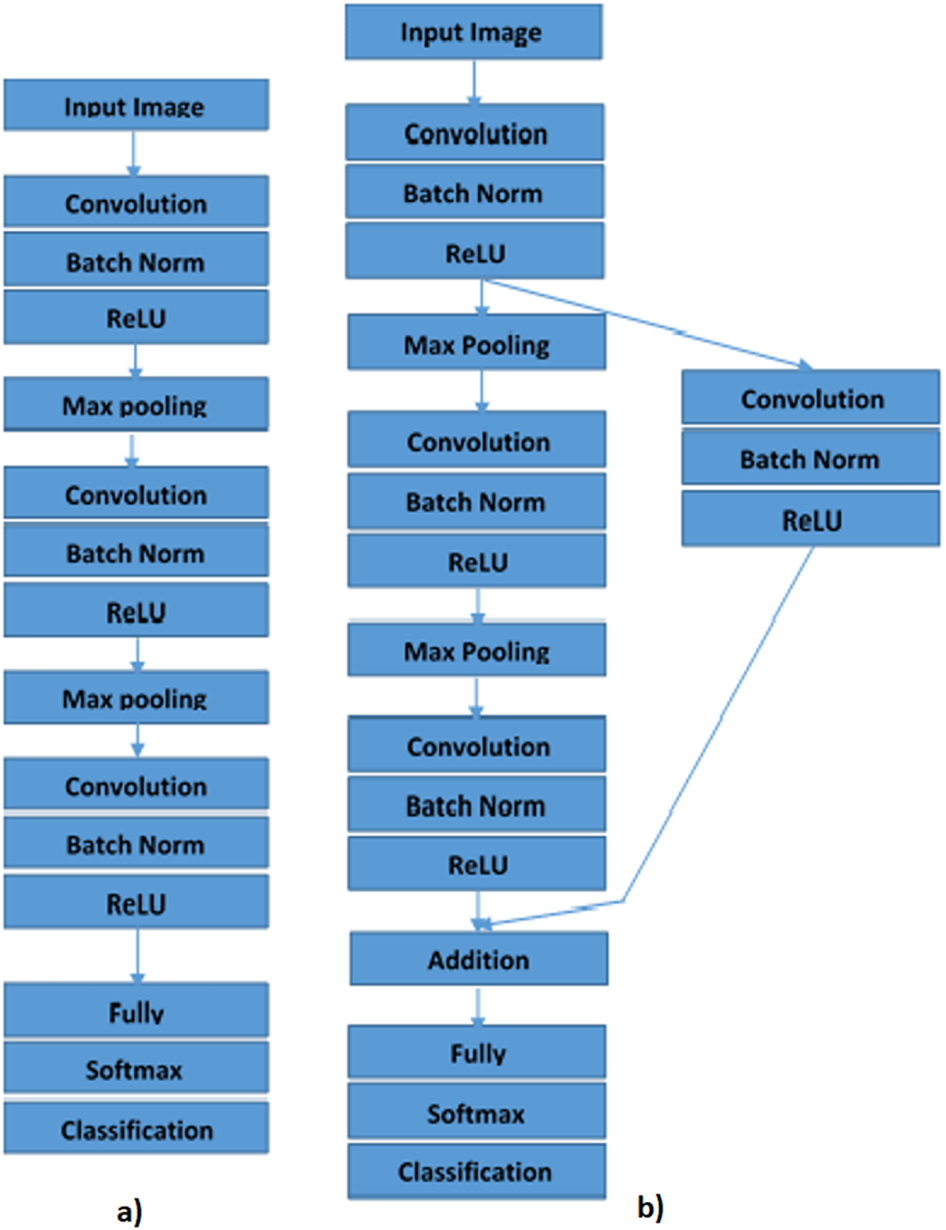
Proposed methods for image classification of Cassava Mosaic Diseases: (A) Plain Convolution Neural Network (PCNN), (B) Deep Residual Neural Network (DRNN).

In this paper, PCNN comprises the image input layer, three convolution layer, three batch normalization layer, three rectified linear units (ReLU), two max-pooling layers, one fully connected layers, one softmax layer, and one classification layer as shown in Fig. 4A. In PCNN, all the layers are connected sequentially (Grohs, Wiatowski & Bölcskei, 2016). The image input size used in this is 30 × 30 × 3. Three convolutional layers have a different filter size, number of filters, and padding. Three convolutional layers utilize the filter size of 3 by 3, while the filter numbers increase from 15 to 30. A padding of 1 ensures that the output has the same size as the input. Batch normalization layers normalize the data propagating over the network to allow the optimization of network training. The ReLU layers improve network training and reduce network sensitivity. The size of the max-pooling layer utilizes in research is 1 this enables us to down-sample the operation.

Down-sampling allows the number of calculations required per layer to be increased without increasing the number of filters. The convolutional layer and batch normalization layers are followed by a fully connected, softmax, and a classification layer. A fully connected layer incorporates all the features of the previous layers and can identify the larger patterns. In this research, the output of the fully connected layer is 5, corresponding to the five classes of cassava disease. The softmax activation function normalizes the performance of the fully connected layer, while the classification layer is used to assign the input to a class that is exclusive to each of these classes to the probabilities returned by the softmax function.

### Deep residual neural network

Deep Residual Neural Network (DRNN) has sequentially connected layers and a shortcut connection with a single convolutional, batch normalization, and ReLU layer (Gurumurthy, Kiran Sarvadevabhatla & Venkatesh Babu, 2017; Wiatowski & Bolcskei, 2015). Shortcut connections make it easier to flow from the output layer to the previous layers of the network through the parameter gradients. Based on PCNN that are sequentially arranged, we insert a shortcut connection that turns the network into a residual network. We create another 1 × 1 convolutional layer, batch normalization, and ReLU layer and add it to the layer graph. We create the shortcut connection from the first ReLU to the additional layer by connecting the first ReLU layer to the fourth convolutional, batch normalization and ReLU layers created. The addition layer sums the output of the third ReLU and the fourth convolutional, batch normalization, and ReLU layers as is shown in Fig. 4B.

### Performance metrics

In literature, the researchers have used different performance metrics to classify images. In this study, eight widely used performance metrics such as accuracy (Ac), sensitivity (Se), specificity (Sp), positive predicted value (PPV), negative predicted value (NPV), area under the curve (AUC), 95% confidence interval (CI) and Kappa (K) are chosen.

## Results and discussion

In this section, we provide the experimental results of our study on Plain Convolution Neural Networks (PCNN) and Deep Residual Neural Networks (DRNN). Throughout the experiments, we employed stochastic gradient descent optimization with a momentum of 0.9 for training. The learning rate and the learning rate drop factor is set to 0.1, while the learning rate drop period is set to 60. We use a total of 80 epochs for the classification of both balanced and imbalanced dataset of cassava mosaic diseases. Tables 2 and 3 show the performance metrics of both PCNN and DRNN on the imbalanced dataset of cassava leaf mosaic disease dataset. Different performance metrics were estimated: specificity (Sp), sensitivity (Se), balanced accuracy (Ab), overall accuracy (Ac), kappa (K), 95% confidence intervals (CI), and area under the ROC curve (AUC).

**Table 2:** Classification performance metrics of PCNN using imbalanced dataset of cassava leaves.

	*S_e_* (%)	*S_p_* (%)	PPV (%)	NPV (%)	*A_b_* (%)
healthy	1.82	99.81	33.33	95.21	50.82
cbb	2.88	99.22	27.27	90.98	51.05
cbsd	47.60	83.19	49.64	82.02	65.40
cgm	2.55	98.77	25.00	86.28	50.66
cmd	86.23	39.14	54.93	76.77	62.69

**Table 3:** Classification performance metrics of DRNN on imbalanced dataset of cassava leaves.

	*S_e_* (%)	*S_p_* (%)	PPV (%)	NPV (%)	*A_b_* (%)
healthy	0.00	100	–	95.14	50.00
cbb	0.00	100	–	90.81	50.00
cbsd	0.00	100	–	74.18	50.00
cgm	0.00	100	–	86.12	50.00
cmd	100	0.00	46.24	–	50.00

The PCNN network applied on the imbalanced dataset has failed to classify cassava mosaic disease as shown by the values of the performance metrics such as Se, Sp, PPV, NPV. The balanced accuracy of the PCNN of the five classes of cassava mosaic disease, for example, healthy, CBB, CBSD, CGM, and CMD is within the range of 50–65% as shown in Table 2. PCNN failed to classify cassava mosaic disease correctly. The balanced accuracy of the PCNN of the five classes of cassava mosaic disease, for example, healthy, CBB, CBSD, CGM, and CMD is within the range of 50–65%. DRNN, on the other hand, also has failed to classify cassava mosaic disease correctly. The balanced accuracy of the DRNN of the five classes of cassava mosaic disease, for example, healthy, CBB, CBSD, CGM, and CMD, is about 50% in all the five classes considered in this paper. This shows that both PCNN and DRNN fail to learn on the imbalanced cassava disease dataset.

Tables 4 and 5 show the values of the classification performance of PCNN and DRNN on the balanced dataset. The accuracy of PCNN is within the range of 75-99%, this shows that PCNN failed to achieve high accuracy of recognition of cassava leaf disease as shown in Table 4. The balanced accuracy of DRNN is within the range of 94–99%, this shows that DRNN performs much better than PCNN as shown in Table 5.

**Table 4:** Classification performance metrics of PCNN on balanced dataset of cassava leaves.

	*S_e_* (%)	*S_p_* (%)	PPV (%)	NPV (%)	*A_b_* (%)
healthy	100	98.19	93.18	100	99.10
cbb	99.49	97.95	92.24	99.87	98.72
cbsd	87.12	94.39	79.31	96.74	90.76
cgm	97.82	95.53	85.02	99.41	96.67
cmd	53.23	98.31	88.80	89.31	75.77

**Table 5:** Classification performance metrics of DRNN on balanced dataset of cassava leaves.

	*S_e_* (%)	*S_p_* (%)	PPV (%)	NPV (%)	*A_b_* (%)
healthy	99.52	99.10	86.77	99.97	99.52
cbb	99.35	99.11	90.88	99.94	99.35
cbsd	98.34	99.14	97.53	99.43	98.34
cgm	99.22	99.05	94.29	99.87	99.22
cmd	94.36	99.75	99.70	95.23	94.36

Comparing the overall performance statistics of the imbalanced and balanced dataset from Tables 6 and 7, DRNN performs better on the balanced dataset, with an overall accuracy of 96.75%.

**Table 6:** Overall leave disease recognition performance on imbalanced dataset of cassava leaves.

	*A_c_* (%)	*K*	95% CI	AUC
PCNN	52.87	0.2112	[0.4992–0.5582]	0.5839
DRNN	46.24	0.00	[0.4331–0.492]	0.5000

**Table 7:** Comparison of leaf disease recognition performance on balanced dataset of cassava leaves.

	*A_c_* (%)	K (%)	95% CI	AUC
PCNN	87.50	84.37	[0.8597–0.8892]	0.9129
DRNN	96.75	95.94	[0.9588–0.9748]	0.9783

**Figure 5:**
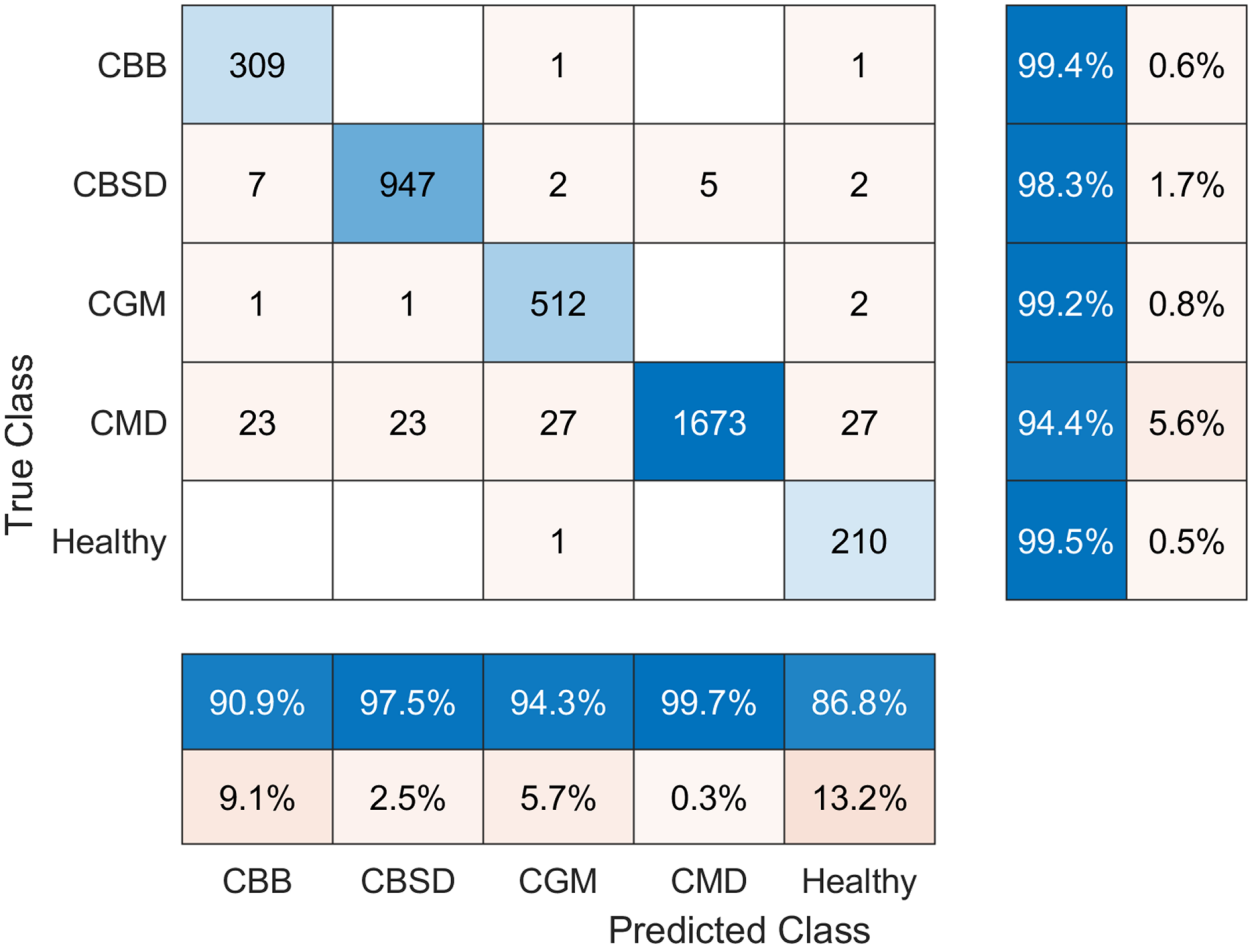
Confusion matrix of balanced cassava leaf dataset classification results using DRNN.

The confusion matrix of the results achieved by DRNN is presented in Fig. 5. Note that the best accuracy was achieved by predicting the Healthy class, while comparatively worst disease recognition results were achieved by predicting the CMD class. Nevertheless, the accuracy is still good (94.4%).

Despite good results, our method also has some limitations. First, all deep learning-based methods tend to overfit the training dataset, which prevents them from generalizing. Also, image enhancement using gamma correction may not be the best method in case of adverse photographing conditions.

## Conclusions

The PCNN and DRNN models were developed for the recognition of cassava leaf diseases. We have adopted the distinct block processing technique that allowed us to counterbalance the original imbalanced dataset of cassava leaf images, which was biased towards CMD and CBSD disease classes. Besides, the DRNN model has produced the best results for our predictive model and achieved the accuracy of 96.75% on the Cassava Disease Dataset from Kaggle. As a result, the technique has proven to be highly effective in classifying cassava leaf diseases. In future work, we will explore novel image augmentation methods combined with other types of deep neural networks (such as Capsule Neural Networks) to further improve the recognition accuracy.

## Supplemental Information

10.7717/peerj-cs.352/supp-1 Supplemental Information 1 MATLAB code of implementation.

## Additional Information and Declarations

### Data Availability

The following information was supplied regarding data availability: We used an openly available dataset from Kaggle: https://www.kaggle.com/c/cassava-disease. MATLAB scripts are available in the Supplemental Files.
